#  Factor VIII replacement prophylaxis in patients with hemophilia A transitioning to adults: a systematic literature review

**DOI:** 10.1186/s13023-021-01919-w

**Published:** 2021-06-26

**Authors:** Jing Sun, Xuan Zhou, Nan Hu

**Affiliations:** 1 Department of Hematology, Nanfang Hospital, Southern Medical University, No. 1838 North Guangzhou Avenue, Guangzhou, 510515 China; 2 Medical Affairs, Pharmaceuticals, Bayer Healthcare Company Ltd, Beijing, 100020 China

**Keywords:** Hemophilia, Prophylaxis, Adherence, Young adults

## Abstract

**Background:**

Despite the advantages of prophylactic treatment for hemophilia, patients tend to discontinue or not adhere to it because of several challenges such as long-term use, high cost, young patients transitioning to adolescents, and switch to self-infusion or self-care. The goal of this systematic literature review is to emphasize adherence to and efficiency of prophylactic treatment in adults.

**Methods:**

A literature review was conducted in PubMed, Embase, and Cochrane databases until April 2021 according to PRISMA guidelines, and the protocol was registered with PROSPERO (CRD42020220085). Studies evaluating the efficacy of prophylaxis in enhancing the quality of life were included.

**Results:**

A total of 31 articles involving 2379 patients with hemophilia were included in this systematic review. Of these, 26 studies were observational, questionnaire-based studies, and 5 were randomized controlled trials. The majority of studies reported lower annualized bleeding rates in patients receiving prophylaxis compared with those receiving on-demand treatment or those who discontinued prophylaxis. Standard-dose prophylaxis was reported to be effective in most of the studies. In developing countries like China, data suggest that low doses were administered because of limited available resources. However, standard dose or individualized prophylaxis should be provided to prevent joint damage in the long term. Compared with adults, greater adherence to treatment was observed in patients aged < 16 years.

**Conclusion:**

This systematic review emphasizes the importance of adherence to prophylaxis among young adults transitioning from childhood. In countries like China, low-dose prophylaxis can help in preventing joint bleeds in the short term, but in the long term, standard-dose therapy has shown high adherence among young adults and better joint health, in turn improving the quality of life.

**Supplementary Information:**

The online version contains supplementary material available at 10.1186/s13023-021-01919-w.

## Introduction

Hemophilia A and B are X chromosome-linked bleeding disorders caused by mutations in factor VIII (FVIII) and factor IX (FIX) genes, respectively [1]. Hemophilia A accounts for 80–85% of all hemophilia cases [2–4]. Consequently, the ability of the blood to coagulate gets impaired, leading to an increased risk of delayed bleeding, which in turn results in serious and life-threatening health problems. It is more frequently observed in males compared with females and may be caused by homozygosity and lionization [1, 5]. On the basis of clotting factor concentrations, the disease can be severe (factor level of < 1 IU/dL), moderate (1–5 IU/dL), or mild (> 5 IU/dL). Patients with severe hemophilia represent about half of diagnosed cases [2, 6, 7].

The common serious sites of bleeding in hemophilia include joints (hemarthrosis), muscles, especially deep compartments (iliopsoas, calf, and forearm), and mucous membranes in the mouth, gums, nose, and genitourinary tract, whereas life-threatening bleeding sites include intracranial, neck/throat, or gastrointestinal regions. The frequency of bleeding varies depending on the site: joints (70–80%), muscle (10–20%), other sites (major bleeds; 5–10%), and central nervous system (< 5%) [2]. The risk of mineral density is high in patients with hemophilia compared with the normal population, which may be due to severity of hemophilia, hemophilic arthropathy, and the resultant immobility. Hence, the World Federation of Hemophilia (WFH) recommends regular physical activity [8].

Hemophilia A has an estimated incidence of approximately 24.6 cases per 100,000 births [8]. According to the WFH Annual Global Survey of 2018, the number of people with hemophilia around the world is approximately 400,000, with India reporting the highest prevalence (20,778), followed by the United States (17,757) and China (14,390) [2–4].

Evidence suggests that prophylaxis with factor replacement drugs, plays a significant role in reducing the number of bleeds per year and prevents joint damage when compared with on-demand treatment. Although approximately 400,000 people globally are affected by hemophilia, only 25% receive adequate treatment [2]. In China, the registration rate for patients with hemophilia is very low, and of the registered patients, only 15% and 7% aged < 18 years and > 18 years, respectively were estimated to be on prophylaxis [4]. The number of patients under prophylaxis is increasing but still low in China. Inadequate treatment of hemophilia can lead to joint damage, which would eventually lead to increased pain, reduced physical activity, necessity of synovectomy, or prolonged bleeding due to injury or surgical procedures or severe bruising [9, 10]. Hence, the goal of hemophilia management guidelines such as WFH, NORDIC, and Chinese is to prevent bleeding and the associated musculoskeletal complications, which restores normal life activities and social participation attained using prophylaxis [5, 8, 11, 12]. The WFH recommends initiation of prophylaxis at any age to reduce hemarthrosis and slow down the progression of hemophilic arthropathy [8]. As per National Hemophilia Foundation (NHF) guidelines, patients with hemophilia benefit from lifelong preventive treatment. British Society for Hematology (BSH) guidelines recommend that adolescents and adults with severe hemophilia should be encouraged to continue regular prophylaxis, and the frequency and dose should be adjusted according to their bleeding phenotype and individual pharmacokinetic (PK) data [13]. In China, the WFH began its intervention in hemophilia care in 1993 and established the Hemophilia Treatment Center Collaborative Network of China (HTCCNC) consisting of 6 core hemophilia treatment centers (HTCs) in 2004 [3, 14]. Now the number of HTCs has expanded to 115. Though standard dose is recommend, due to limited factor availability and high costs, HTCCNC conducted studies to show benefit of low dose prophylaxis [14].

Prophylactic treatments mainly include treatment by intravenous injection of factor concentrate. The protocols for prophylactic treatments are classified, depending on the situation, as episodic (on-demand treatment, at the time of clinically evident bleeding), intermittent (periodic, treatment given to prevent bleeding for periods not exceeding 45 weeks in a year), and continuous (primary, secondary, and tertiary prophylaxis) [2]. Although prophylaxis is a gold standard of care for treating severe hemophilia, its use is limited by issues such as patient’s age, physical activity, lifestyle, joint status, ways of assimilating inconsistency in bleeding phenotype, dosing levels, dosing intervals, individual response to factor concentrate, and adherence to treatment. Individualized prophylaxis can be an alternative approach that helps to address these issues [15].

Adherence to treatment recommendations and follow-up is an ongoing challenge common to long-term medical conditions. Data suggest that up to 20% to 87% of patients with hemophilia do not follow the prescribed treatment [16–19]. Despite the advantages of prophylactic treatment for hemophilia, patients tend to discontinue or not adhere to it because of challenges such as long-term use, high cost, young patients transitioning to adolescents, and switch to self-infusion or self-care. In China, national reimbursement drug list (NRDL) does not provide financial assistance for adult prophylaxis which may lead to discontinuation of prophylaxis in patients aged > 18 years. The WFH recommends age-specific hemophilia camps to understand the importance of adherence to prophylaxis and develop self-infusion skills. Despite multiple national guidelines, clinical evidence elucidating the outcomes of non-compliance to prophylactic treatment in hemophilia patients transitioning to adolescents is limited. Hence, there is an unmet need for designing transition therapy.

The present systematic literature review therefore summarizes factors (barriers and promoters) for adherence to prophylactic treatment in young adults, the effect of non-adherence to prophylaxis, the importance of prophylaxis in young adults, and the effect of different doses (low, medium, and standard dose) and durations of treatment (short and long term) on patients’ health.

## Methodology

**Fig. 1:**
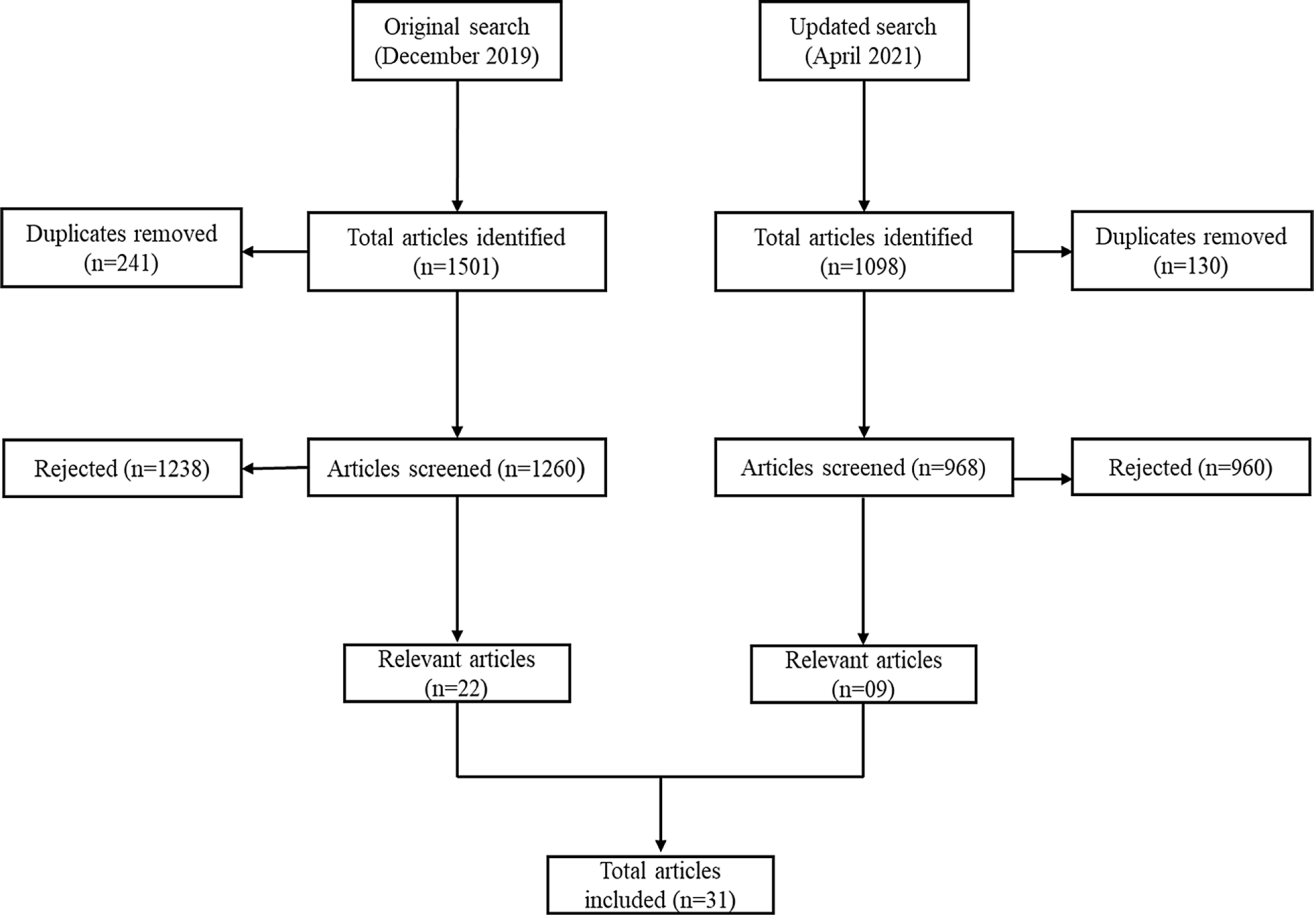
Prisma flowchart

A systematic literature review was performed as per the Preferred Reporting Items for Systematic Reviews and Meta-Analyses (PRISMA) guidelines (Fig. 1) [20] and was registered with PROSPERO (CRD42020220085). We searched PubMed, Embase, and Cochrane databases until December 2019. The search was updated till April 2021. The following search strategy was used to identify articles: 'hemophilia'/exp AND ('adolescent'/exp OR 'child'/exp) AND ('patient compliance'/exp OR 'medication compliance'/exp OR 'quality of life'/exp OR 'self care'/exp OR 'self concept'/exp OR 'transition to adult care'/exp OR ‘adult’/exp). Two reviewers independently screened the abstracts and full texts for eligibility of inclusion. Studies [randomized clinical trials (RCTs), original and observational studies] published in English that evaluated the efficacy of prophylaxis in improving quality of life were considered eligible. Articles reporting insufficient data, studies published in languages other than English, articles on non-factor replacement, and articles on topics other than prophylaxis were excluded from the study.

('hemophilia'/exp) AND ('adolescent'/exp OR 'child'/exp) AND ('patient compliance'/exp OR 'medication compliance'/exp OR 'quality of life'/exp OR 'self care'/exp OR 'self concept'/exp OR 'transition to adult care'/exp OR ‘adult’/exp).

(hemophilia[Title/Abstract]) AND ((adolescent[Title/Abstract]) OR (child[Title/Abstract]) OR (adult[Title/Abstract])) AND ((patient compliance[Title/Abstract]) OR (medication compliance[Title/Abstract]) OR (quality of life[Title/Abstract]) OR (self-care[Title/Abstract]) OR (self-concept[Title/Abstract]) OR (transition to adult care[Title/Abstract])).

Studies on prophylactic factor replacement therapies such as FVIII in patients with hemophilia A were included. Studies published in languages other than English, studies on prophylaxis using non-factor treatments, reviews, and meta-analyses were excluded.

Duplicates and articles not meeting the eligibility criteria were removed and disagreement was resolved by a third reviewer. The following data from each study were collected: (1) prophylaxis treatment, (2) study type, (3) number of patients, (4) age of patients, (5) follow-up period, and (6) outcomes measured. The data were segregated on the basis of dose of FVIII used for prophylaxis and duration of prophylaxis. The methodological quality was assessed using Jadad scale for RCTs and Newcastle Ottawa scale for non-RCTs.

### **WFH definitions **[8]

Primary prophylaxis: “Regular continuous prophylaxis started in the absence of documented joint disease, determined by physical examination and/or imaging studies, and before the second clinically evident joint bleed and 3 years of age.”

Secondary prophylaxis: “Regular continuous prophylaxis initiated after 2 or more joint bleeds but before the onset of joint disease; this is usually at 3 or more years of age.”

Tertiary prophylaxis: “Regular continuous prophylaxis initiated after the onset of documented joint disease. Tertiary prophylaxis typically applies to prophylaxis commenced in adulthood.”

## Results

The search of the database yielded 2469 results after removing duplicates, of which 31 articles with a total of 2379 patients with hemophilia were included in the present systematic review. Almost all studies were on severe hemophilia. The methodological quality of included non- RCTs was done using Newcastle Ottawa scale was represented in Additional file 1: Table S1. Of the 30 studies (Table 1), the majority (86.7%) were observational studies and 5 were RCTs. Eighteen studies (58.1%) studies reported dosage of FVIII administered and 15 (50.0%) studies reported the duration of treatment (Table 2).

**Table 1:** Characteristics and quality assessment of studies included

SL. no	Study title	Author (s)	Year	Study design	Type of treatment	Age	Quality assessment^a^
*Articles on non-Chinese patients*							
1	Can long-term prophylaxis for severe haemophilia be stopped in adulthood? Results from Denmark and the Netherlands [7]	van Dijk	2005	Observational	Discontinuation of prophylaxis	Denmark: 26.2 (23.8–29.1) years Netherlands: 26.5 (23.9–29.5) years	6
2	A comparison between prophylaxis and on demand treatment for severe haemophilia [21]	Khoriaty	2005	Observational	Prophylaxis vs on-demand	Mean age: 27.93; median age: 28 years	4
3	A randomized clinical trial of prophylaxis in children with hemophilia A (the ESPRIT Study) [22]	Gringeri	2011	RCT	Prophylaxis vs episodic treatment	50 (10–84); 48 (14–84) years	3
4	A randomized comparison of two prophylaxis regimens and a paired comparison of on-demand and prophylaxis treatments in hemophilia A management [23]	Valentino	2012	RCT	Prophylaxis vs on-demand	26 years (7–59) years	3
5	Consequences of switching from prophylactic treatment to on-demand treatment in late teens and early adults with severe haemophilia A: the TEEN/TWEN study [24]	Manco-Johnson	2013	Prospective	Prophylaxis vs prospective on-demand vs retrospective on-demand	19.5 (13–31) years	5
6	Controlled, cross-sectional MRI evaluation of joint status in severe haemophilia A patients treated with prophylaxis vs on demand [25]	Oldenburg	2015	Cross-sectional	Prophylaxis vs on-demand	12–16; 17–21; 22–26; 27–35 years	5
7	Prophylaxis vs. on-demand treatment with BAY 81–8973, a full-length plasma protein-free recombinant factor VIII product: results from a randomized trial (LEOPOLD II) [26]	Kavakli	2015	RCT	2 low-dose and 2 high-dose prophylaxis vs 2 on-demand	12–65 years	3
8	Adherence to prophylaxis and quality of life in children and adolescents with severe haemophilia A [17]	García-Dasí	2015	Cross-sectional	Prophylaxis	6–20 years	5
9	Adherence to clotting factors among persons with hemophilia A or B [27]	Armstrong	2015	Retrospective	Prophylactic	Range: < 1 to 61 years	5
10	Benefits of prophylaxis versus on-demand treatment in adolescents and adults with severe haemophilia A: The POTTER study [28]	Tagliaferri	2015	Prospective	Prophylaxis vs on-demand	12–55 (12–25 and 26–55) years (grouping based on age)	6
11	Adherence to treatment regimen and bleeding rates in a prospective cohort of youth and young adults on low-dose daily prophylaxis for severe hemophilia A [29]	Mizrahi	2016	Prospective, longitudinal	Low-dose prophylaxis	15.2–28.4 years	6
12	Objective quantification of adherence to prophylaxis in haemophilia patients aged 12 to 25 years and its potential association with bleeding episodes [30]	Pérez-Robles	2016	Retrospective	Prophylaxis	Range: 12–15; mean age: 17.56 years	4
13	Discontinuing early prophylaxis in severe haemophilia leads to deterioration of joint status despite low bleeding rates [31]	Nijdam	2016	Observational	Prophylaxis	15.3 years	6
14	Adherence to prophylaxis and bleeding outcome in haemophilia: a multicentre study [32]	Schrijvers	2016	Prospective	Adherence to prophylaxis	Parent-reported age: 8.4 (6.2–10.5) years; patient-reported age: 29.9 (17.1–49.8) years	4
15	Effect of late prophylaxis in hemophilia on joint status: a randomized trial [33]	Manco-Johnson	2017	RCT	Prophylaxis vs on-demand	12–50 years	2
16	Tailored frequency-escalated primary prophylaxis for severe haemophilia A: results of the 16-year Canadian Hemophilia Prophylaxis Study longitudinal cohort [34]	Feldman	2018	Longitudinal	Prophylaxis	1–2.5 years	5
17	Young adult outcomes of childhood prophylaxis for severe hemophilia A: Results of the joint outcome continuation study [35]	Warren	2020	Observational, partially retrospective	Prophylaxis	–	6
18	Long-term analysis of the benefit of prophylaxis for adult patients with severe or moderate haemophilia A [36]	Miesbach	2020	Prospective, noninterventional, multicenter, binational, long-term	Prophylaxis	–	6
19	Intermediate dose prophylaxis in adults with haemophilia: a clinical audit from a resource limited setting [37]	Sudevan	2020	Clinical audit	Prophylaxis vs on-demand	31.63 ± 6.98 years	5
20	Physical activity improved by adherence to prophylaxis in an Italian population of children, adolescents and adults with severe haemophilia A: the SHAPE study [38]	Zanon	2020	Prospective	Prophylaxis	< 12 years; 12–18 years and > 18 years	6
21	Hemophilia prophylaxis adherence and bleeding using a tailored, frequency-escalated approach: the Canadian Hemophilia Primary Prophylaxis Study [39]	Dover	2020	Observational	Prophylaxis	12–30 months	5
22	The perspectives of adolescents and young adults on adherence to prophylaxis in hemophilia: a qualitative study [40]	Hoefnagels	2020	Qualitative	Prophylaxis	Median 18 (14–25) years	6
23	Prophylactic vs episodic treatment to prevent bleeds and preserve joint function in Thai children with moderate and severe haemophilia A [41]	Songnuy	2020	Prospective cohort	Episodic vs prophylaxis	≥ 6 months to ≤ 18 years	5
*Articles on Chinese patients*							
1	Low-dose tertiary prophylactic therapy reduces total number of bleeds and improves the ability to perform activities of daily living in adults with severe haemophilia A: a single-centre experience from Beijing [42]	Hua	2016	Retrospective	Prophylaxis	18–60 years (median 31)	6
2	A prospective study of health-related quality of life of boys with severe haemophilia A in China: comparing on-demand to prophylaxis treatment [43]	Wu	2017	Prospective	Prophylaxis Vs on-demand	4–15.9 years	5
3	Long-term efficacy and safety of prophylaxis with recombinant factor VIII in Chinese pediatric patients with hemophilia A: a multi-center, retrospective, non-interventional, phase IV (ReCARE) study [44]	Li	2017	Retrospective	Prophylaxis	7.1 ± 4.23 years	5
4	Describing the quality of life of boys with haemophilia in China: Results of a multicentre study using the CHO-KLAT [45]	Tang	2017	Cross-sectional	Both hemophilia A and B; Prophylaxis or on-demand	4 to 17.9 years; median: 8.4 years	4
5	Efficacy of short-term full-dose prophylaxis in adult Chinese patients with severe hemophilia A [46]	Sun	2018	Prospective	Prophylaxis Vs on-demand	26 (20.5–29.0) years	6
6	Efficacy of Short- term Individualized Prophylaxis Guided by PK and Joint Evaluation in Chinese Adult Patients with Severe Hemophilia A [47]	Sun	2019	Prospective	Prophylaxis Vs on-demand	–	
7	Efficacy and safety of prophylaxis with BAY 81‐8973 in Chinese patients with severe haemophilia A enrolled in the LEOPOLD II trial [48]	Yang	2019	RCT	Low-dose Vs high-dose Vs on-demand	12–65 years	3
8	Long-term joint outcomes of regular low-dose prophylaxis in Chinese children with severe haemophilia A [49]	Wu	2021	Retrospective	Prophylaxis	–	6

RCT, Randomized clinical trial

^a^Quality assessment was done using Jadad scale (0–5) for RCTs and Newcastle Ottawa scale (0–8) for non-RCTs

**Table 2:** Dose, duration of treatment, and clinical outcomes of studies included

Study	n	Type of prophylaxis	Dose	Duration of treatment	Clinical outcomes	
					Prophylaxis	On-demand
*Articles on non-Chinese patients*						
van Dijk et al. [6, 7]	22 Danish 58 Dutch	–	–	Long-term	AJBR: 1.8 (0.0–3.0) Pettersson score: 13.0 (5.0–23.0)	AJBR: 3.2 (0.9–6.0) Pettersson score: 13.0 (0.8–23.5)
Khoriaty [21]	133	–	–	–	AJBR: 3.2 ± 6.4	AJBR: 5.7 ± 9.1
Gringeri [22]	40	–	Medium	Long-term	≤ 3 y: (0.35 events/patient/month); joint bleeds (0.12 events/patient/month) 3 y at the start of prophylaxis: (0.62 and 0.25 events/patient/month) ≤ 3 y: 3 bleeds/year	–
Valentino [23]	53	–	Standard	Long-term	AJBR: Standard dose: 1.6 ± 1.2; Pharmacokinetic tailored dose: 1.9 ± 1.1	–
Manco-Johnson et al. [24]	38	–	Standard	–	Joint pain: 0.6 ± 0.9 AJBR: 0	AJBR: Discontinued prophylaxis ≤ 12 months before study: 4.8; Discontinued prophylaxis ≥ 13 months before study: 24
Oldenburg [25]	118	Primary and secondary	–	–	Mean index joint score: Secondary prophylaxis: 7.5 (0–10.5) MRI score: Secondary prophylaxis: 16.5 (0–20) Joint bleeds during previous 5 years: All prophylaxis, ≤ 4.1	Mean index joint score: 13.8 (0.5–19.5) MRI score: 18 (2–19) Joint bleeds during previous 5 years: ≤ 14.3
Kavakli [26]	80	–	Standard	–	AJBR: 4.9 ± 6.8	AJBR: 57.7 ± 24.6
García-Dasí [17]	78	Primary or secondary	–	Long-term	–	–
Tagliaferri [28]	53	Tertiary	Medium	Long-term	AJBR: 12–25 y (1.97); 26–55 y (2.46) Pettersson score: 12–25 y: 5.5 (4.9); 26–55 y: 22.2 (18.5)	AJBR: 12–25 y (16.8);26–55 y (16.71) Pettersson score: 12–25 y: 5.7 (6.7); 26–55 y: 35.0 (17.2)
Mizrahi et al. [29]	25	–	Low	–	HJHS score: 16	–
Pérez-Robles et al. [30]	52	Primary or secondary	Standard	–	–	–
Nijdam et al. [31]	66	–	Medium	Long-term	HJHS score: discontinued (23); continued (14) AJBR: discontinued (1.5); continued (1.2)	–
Schrijvers et al. [32]	241	–	–	–	AJBR: Children (3.7); adult (3)	–
Manco-Johnson et al. [33]	70	Tertiary	–	Long-term	Joint pain: At 3 y, 50% decrease in pain for the previous 4 weeks ([− 17.2 ± 22.9]; − 16.0 [− 75 to 21]) MRI score: LS mean from baseline to 3 years: 0.79 HRQoL: At 3 years, improved by 3.98 points AJBR: 27.3 (14.9–41.1); 28.7 ± 18.8	Joint pain: At 3 y, reported no change ([0.0 ± 25.1); − 3.0 (− 52 to 52]) MRI score: LS mean from baseline to 3 years: 0.96 HRQoL: At 3 years, deterioration by 6.00 points AJBR: 0.3 (0–1.2); 1.9 ± 4.1
Feldman et al. [34]	56	Primary	Tailored frequency-escalated	Long-term	AJBR: Index hemarthroses: 0.95 (0.44–1.35; 0.00–13.43) Other hemarthroses: 0.11 (0.00–0.30; 0.00–1.76)	–
Warren et al. [35]	37	Primary and tertiary	–	Long-term	MRI score: Primary prophylaxis, 1.3 (05–2.2) vs tertiary prophylaxis, 2.3 (1.3–5.0) AJBR: Primary prophylaxis, 0.8 (0–3) vs tertiary prophylaxis, 0.4 (0–3); At age 6 years: Primary prophylaxis, 0.7 ± 0.8 vs tertiary prophylaxis, 3.8 ± 2.8	–
Miesbach et al. [36]	161	–	–	Long-term	AJBR: Median: 1.3 (IQR: 3.6)	AJBR: Median 31.4 (IQR: 27.6)
Sudevan et al. [37]	8	Tertiary	Medium	Short-term	AJBR: 0.63 ± 0.74/month	AJBR: 5.13 ± 2.51/month
Zanon et al. [38]	42	–	Standard	Long-term	HJHS score: Baseline: 0.1 ± 0.4; post follow-up: 2.3 ± 3.2	–
Dover et al. [39]	56	–	Individualized	Long-term	MRI score: 20.27 ± 12.36 AJBR: 3.71 ± 1.35	–
Songnuy et al. [41]	15	–	Medium	Long-term	HRQoL: 85.71 ± 8.52 HJHS score: 9.17	HRQoL: 72.86 ± 10.87 HJHS score: 12.75
*Articles on Chinese patients*						
Hua et al. [42]	33	Tertiary	Low	–	AJBR: 11.8 ± 7.6	AJBR: 41.5 ± 20.4
Wu et al. [43]	23	–	Standard	Short-term	Mean CHO-KLAT score: Child self-reported, 61.9 ± 11.4; Parent proxy reported, 58.2 ± 8.1	Mean CHO-KLAT score: Child self-reported, 61.4 ± 10.9; Parent proxy reported, 54.4 ± 10.5
Li et al. [44]	183	–	–	–	AJBR: 4.91 (8.110); ABR: 8.44 (10.892)	–
Tang et al. [45]	269	–	–	–	HRQoL: Child self-reported: 58.9 ± 15.6 (range 28.1–96.9); parent proxy-reported: 51.9 ± 14.9 (range 16.7–100)	–
Sun et al. [46, 47]	27	Individualized and standard	Medium	–	–	–
Yang et al. [14, 48]	80	–	Standard		Median ABRs: 2.0 (Chinese); 1.0 (Non-Chinese patients)	Median ABRs: 61.3 (Chinese); 58.5 (Non-Chinese patients)
Wu et al. [49]	21	–	Low	Long-term	MRI score: Total: 2–24 HJHS score: 2–27, with 0–10 for 46.7% children and > 10 for 53.3%	–

MRI, Magnetic Resonance Imaging; HRQoL, Health Related Quality of Life; HJHS, Hemophilia Joint Health Score; AJBR, Joint Annualized Bleeding Rate; ABR, Annualized Bleeding Rate; IQR, Interquartile range

### Adherence to treatment

Several studies reported that discontinuing prophylaxis is a common phenomenon in adulthood [29, 31, 32, 40]. The adherence to prophylaxis was reported to be higher in patients below 16 years of age, which means that patients tend to discontinue prophylaxis while transitioning from childhood to adolescence [17, 21]. Pérez-Robles et al. reported higher adherence at 12 to 25 years of age, in agreement with the study by Schrijvers et al. that reported lower adherence in patients aged 25 to 40 years [30, 32]. Hoefnagels et al. reported decreased parenteral support and bleeding that has less impact on daily life as important factors for non-adherence to prophylaxis [40]. An Italian study reported high adherence to prophylaxis (80%) and increased physical activity in adherent patients [38]. Furthermore, these studies suggested that adherence to prophylaxis reduced with age from childhood to adolescence to adulthood.

### Type of prophylaxis

Based on initiation time of prophylaxis, it is divided into primary, secondary, and tertiary prophylaxis. Among the studies included, 6 studies (2 studies on primary prophylaxis [25, 34] and 4 studies on tertiary prophylaxis [28, 33, 37, 42]) reported the type of prophylaxis given. Two studies were based on either primary or secondary prophylaxis [17, 30], whereas in 1 study both primary and tertiary prophylaxes were followed [35]. From the studies, primary prophylaxis reported better results in terms of joint health outcome and long-term health-related quality of life (HRQoL).

### Effect of prophylactic dose

Three different doses of FVIII are reported to be used for treatment: low (10–15 IU FVIII/kg 2–3 days per week or 1000–1500 IU/kg per year), medium (15–25 IU FVIII/kg 3 days per week or 1500–4000 IU/kg per year), and standard (25–40 IU FVIII/kg every 2 days or > 4000 IU/kg per year). Of the 17 studies reporting dosage, 7 studies reported standard-dose treatment [23, 24, 26, 30, 38, 43, 48] and 3 studies reported low doses [29, 42, 49]. In 5 studies, tailored prophylaxis was used [23, 34, 39, 46, 47]. Among the studies included, the dose of FVIII varied from as low as 5 IU/kg to 50 IU/kg. According to the studies, standard dose is preferred to low dose and tailored prophylaxis is better than standard dose. Although standard dose is preferred, some studies have reported better outcomes even with low doses compared with on-demand treatment [26, 29, 42, 44].

### Effect of duration of prophylaxis

Prophylaxis can vary in terms of the duration of treatment: short-term prophylaxis (1–3 months) and long-term prophylaxis (continuous treatment). Of the studies included, 2 studies reported short-term prophylaxis [37, 43], whereas 14 studies reported long-term prophylaxis [7, 17, 22–24, 28, 31, 34–36, 38, 39, 41, 49]. Long-term prophylaxis resulted in better outcomes than short-term prophylaxis and also showed the need for continuing treatment at adolescence and adulthood. A Chinese study reported the effectiveness of full-dose prophylaxis in the short term in reducing bleeding rates and also partially preventing the progression of joint damage [46].

The majority of studies reported annualized bleeding rates (ABRs) varying from zero joint bleeds to 11.8 bleeds (Table 2). All the studies have reported better ABRs, joint bleed rates, HRQoL with prophylaxis compared with on-demand treatment. The HRQoL was measured by CHO-KLAT scores. The scores were reported to be better with prophylaxis than with on-demand treatment.

## Discussion

The present systematic literature review discusses the need for adherence to treatment by young adults transitioning from childhood to adolescents. The WFH recommends prophylaxis over episodic treatment. Prophylaxis can be initiated before or after onset of joint bleeding and joint disease, but to ensure best efficacy, it should be started as early as possible prior to joint bleeding. Early initiation of prophylaxis also helps in reducing the risk of intracranial hemorrhage [8]. The Medical and Scientific Advisory Council of the National Hemophilia Foundation also recommends prophylaxis at a young age prior to onset of bleeding [50].

In the United States, the percentage of hemophilia treatment centers (HTCs) prescribing primary prophylaxis had significantly increased from 19% in 2003 to 28% in 2005, whereas secondary prophylaxis remained stable and on-demand treatment was observed to decrease. A similar pattern was observed in the United Kingdom where primary prophylaxis increased from 30% in 2003 to 38% in 2005 [51].

In relation to prophylaxis treatment, two groups of patients have to be addressed: adult hemophilia patients who started primary or secondary prophylaxis and maintained good joint health into adulthood and adult hemophilia patients with advanced joint arthropathy and on tertiary prophylaxis [25]. Studies indicated that primary prophylaxis is better than tertiary prophylaxis [22, 33]. The SPINART study showed the importance of primary prophylaxis over secondary prophylaxis. In patients on secondary prophylaxis, joint damage caused in early adulthood was reported to progress [33]. However, secondary prophylaxis can slow joint deterioration and improve quality of life in adolescents and adults compared with on-demand treatment [23]. Oldenburg et al. also reported better results with primary prophylaxis compared with secondary prophylaxis. The study reported better MRI scoring with primary prophylaxis followed by secondary prophylaxis initiated at < 6 years of age. Gringeri et al. also reported fewer joint bleeds (0.12 joint bleeds/patient/month) and no radiological signs of arthropathy with primary prophylaxis [22]. A study in China reported that 90% of boys (aged 6–9 years) with hemophilia had arthropathy [52]. From the literature, adult patients who started primary or secondary prophylaxis at a young age before joint damage have to continue prophylaxis throughout their lives [25, 28, 33, 52].

If prophylaxis is not initiated at a young age and adolescent and adult hemophilia patients show evidence of joint damage, WFH recommends initiation of tertiary prophylaxis and should continue for long term to reduce the number of hemarthroses, spontaneous and breakthrough bleeding, and slow down the progression of hemophilic arthropathy [8]. The POTTER study reported the safety and efficacy of long-term tertiary prophylaxis in adolescents and adults with severe hemophilia [28]. The results indicated a significant reduction in joint bleeds and delay in hemophilic arthropathy. The study also reported no target joints in 67% of patients (18 of 27) in the prophylaxis group and 19% (n = 5) in the on-demand group; 60% of patients were reported to be adherent to prophylaxis [28]. It is evident from the studies that tertiary prophylaxis reduces the total number of joint bleeds and delays the progression of joint damage [28, 33].

**Fig. 2:**
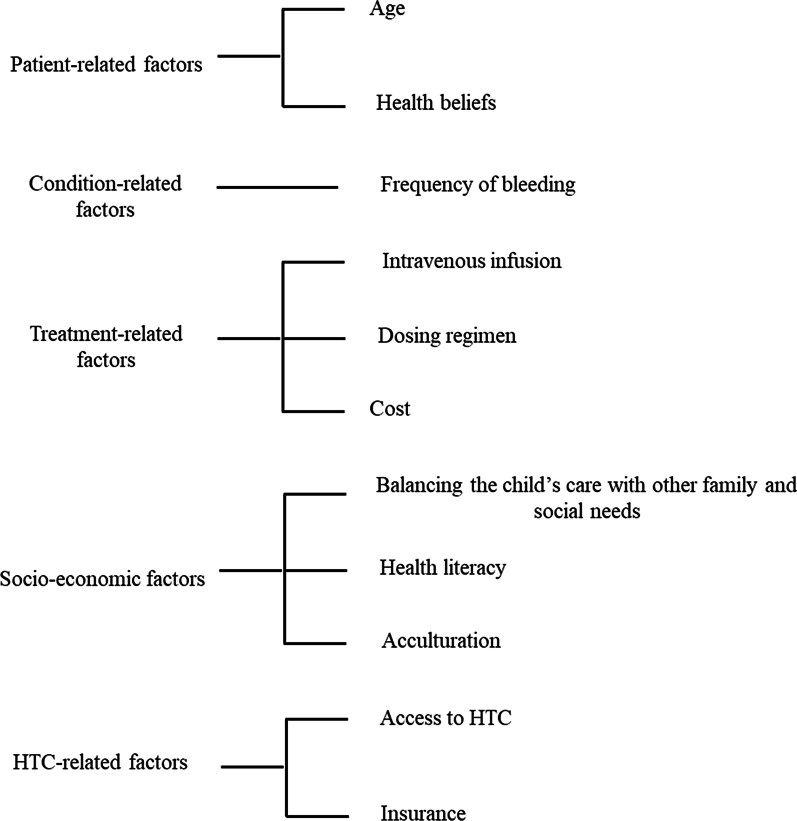
Barriers to treatment adherence. HTC, Hemophilia Treatment Centers

Discontinuation of prophylaxis is commonly observed in young adults transitioning from childhood, which may be due to transitioning from parent care to self-care, moving away from home with increased independence, and high cost of treatment [8, 24]. The barriers to treatment adherence include patient-related factors, condition-related factors, treatment-related factors, and healthcare and socioeconomic factors (Fig. 2). The most frequently reported reasons for non-adherence to prophylaxis are reduction, fluctuation, or disappearance of symptoms, forgetfulness, lack of time, difficulties with self-treatment, and challenges in communicating with HTCs to receive optimal care [53]. On the contrary, Pérez-Robles et al. reported no effect of age, duration of treatment, and prophylactic regimen on adherence [30].

VERITAS-Pro (Validated Hemophilia-Regimen Treatment-Adherence Scale-Prophylaxis) was developed to measure adherence to hemophilia treatment. A US study evaluating adherence to prophylaxis using VERITAS-Pro reported better adherence among pediatric patients (score of 38) compared with adults (score of 45.8) [54]. In accordance with this result, a study conducted in the United States, Canada, and Australia reported scores of 39.6 and 50.8 among children and adults, respectively [18]. In an Spanish study, mean adherence rate was reported as 82.5% using VERITAS-PRO questionnaire [55].

Adult patients on continuous prophylaxis reported better physical functioning and HRQoL than adults who receive limited or no prophylaxis. Children aged < 12 years showed higher adherence to prophylactic treatment than adolescents aged 12–18 years [54]. The TEEN/TWEN study among late teens and young adults reported increased bleeding and worsened HRQoL [24]. Similar results were observed by Fischer et al., but this study provided insufficient information on the long-term effects of stopping prophylaxis permanently in adulthood [56]. Nijdam et al. observed long-term (10-year) effects of discontinuing prophylaxis [31]. The patients developed significant arthropathy compared with patients on prophylaxis [31]. In contrast, van Dijk et al. conducted a study on the long-term effects of discontinuation of prophylaxis in adulthood and showed a low number of joint bleeds and little change in arthropathy after 4 years [7]. Patients with severe hemophilia were found to be more adherent to prophylaxis than those with mild or moderate hemophilia [27]. Zanon et al., reported reduced in number of patients with at least one bleeding episode/year in adherent patients after 3-year follow-up. The study also reported more physical activity with medium impact on joints in highly adherent patients compared to non-adherent patients [38]. For adolescents to adhere to prophylaxis, WFH has recommended to educate adolescents on self-infusion, information on hemophilia to ensure adequate knowledge about the disease, and age-specific hemophilia camps to make them understand the importance of adherence to treatment [8].

Different doses are recommended for prophylaxis [8]. The results of a study by Zhuang et al. comparing low- and intermediate-dose prophylaxis with on-demand treatment indicated that prophylaxis was better than on-demand treatment; however, the prophylaxis doses used did not prevent or reverse the progression of joint damage [57]. Fischer et al. compared intermediate-dose with high-dose prophylaxis and found reduced joint bleeding among patients receiving high doses and on long-term treatment [58]. Hence, of all the doses standard dose is reported to be the most effective dose for prophylaxis [43]. In developing countries like China, economic constraint and shortage of factor concentrates are the major challenges to prophylactic adherence [42]. To overcome this, Hua et al. and Li et al. studied the effects of low-dose prophylaxis and suggested that even low-dose prophylaxis can reduce ABR and improve the quality of life compared with episodic treatment [42, 44]. A study by Andrawes et al. reported that low-dose prophylaxis in severe hemophilia preserves bone mineral density (BMD), increases vitamin D levels, and decreases hemophilia joint health score (HJHS) [59]. Kavakli et al. conducted a study on low- and high-dose prophylaxis; the results of the first 6 months indicated lower ABRs with high doses, but ABRs with both doses were comparable in the next 6 months [26]. A study by Wu et al. reported that long-term use of low-dose prophylaxis resulted in joint damage in all patients and that 81% of patients (17 of 21) had bone defects [60]. The magnetic resonance imaging (MRI) score was reported as 13–24 in 52% of patients (11 of 21). The higher the MRI score the higher the number of joint hemorrhages. A study on long-term joint health outcomes with regular low-dose prophylaxis among Chinese pediatric patients with severe hemophilia A reported better joint outcomes compared with on-demand treatment. The study also reported a certain degree of joint damage in all patients on low-dose prophylaxis, indicating the need for standard dose [49]. Hence, low-dose prophylaxis can be an alternative to standard-dose prophylaxis, especially in developing countries like China for short-term joint preservation but it leads to more frequent clinical and subclinical joint bleeding and joint damage in the long term than standard-dose therapy.

Standard-dose prophylaxis preserves joints, helps in greater adherence, and improves quality of life; hence, it remains the primary option in China [44]. Escalating the frequency of prophylaxis (initiating treatment with a less intense dose [once a week] and gradually increasing the frequency of dosing) helps in improving adherence to treatment as the patients and families gradually adapt to the treatment. This also results in reduced use of central venous access devices. But care and follow-up of patients on less intense doses are required because of a high risk of bleeding until dose escalation is initiated [8]. In a study by Feldman et al., tailored frequency-escalated prophylaxis was studied in children aged 1.0 to 2.5 years who were followed for a maximum of 16.1 years. The study started with a low dose (50 IU/kg once weekly), which was increased to 25 IU/kg on alternate days based on the breakthrough bleeds. The alternate-day prophylaxis was initiated at a median age of 9.9 years. The study reported very little arthropathy and very good health outcomes with tailored frequency-escalated prophylaxis and use of moderate amount of clotting factor compared with standard prophylaxis protocols [34]. Dover et al. also studied tailored frequency-escalated prophylaxis, in which the patients were given prophylaxis at three different dose regimens based on the bleeds. The study started with a low dose of 50 IU/kg of body weight once a week, which was increased to 30 IU/kg of body weight twice a week and further to 25 IU/kg of body weight thrice a week based on the breakthrough bleeds [39].

The WFH recommends escalation of prophylaxis with measurement of trough levels and orthopedic interventions, if required, to patients experiencing breakthrough bleeds even after adhering to their prescribed prophylaxis regimen. Previously, a trough factor level of 1 IU/dL (1%) was considered to be an adequate goal. But it was recognized that patients remain at risk of bleeding with 1% trough level and recent studies showed less bleeding with higher trough levels. Therefore, clinicians are targeting to maintain higher trough levels (> 3% to 5%, or higher). However, to achieve higher trough levels, a higher dose or more frequent infusions of clotting factor may be required. Hence, personalized doses based on an individual’s activities, lifestyle, and PK handling of factor may help in reducing bleeds [8].

Sun et al. compared short-term full-dose prophylaxis and individualized prophylaxis. From the results, PK-tailored individualized prophylaxis led to zero bleeding and no joint damage in adults [47]. For individualized treatment, physicians should consider the bleeding pattern, treatment intensity, and joint health status. Compared with standard-dose prophylaxis, tailored frequency-escalated prophylaxis leads to very little arthropathy and very good health outcomes. It also uses lesser amount of clotting factor compared with standard dose, thereby lowering the cost of treatment and overuse of FVIII [34, 61]. Several predisposing factors such as frequent episodes of immobility, lack of weight-bearing exercises, and comorbidities associated with bone loss lead to decreased BMD in patients with hemophilia. Prophylaxis was reported to preserve BMD, whereas patients on on-demand treatment never attained peak bone mass [62, 63].

### Global experience and its implications for future practice in China

We reviewed adherence to prophylaxis in patients with hemophilia at different ages along with effectiveness of different doses and duration of prophylaxis. Of the 31 studies included, 8 studies are from China. From the results, adherence to treatment was lower in young adults and adults compared with children, which may be due to lack of disease awareness, transitioning from adult care to self-care, and availability of factor. A similar pattern was observed in China. On the other hand, some studies from European countries showed a higher adherence rate, which may be due to availability of factor, time spent at HTCs, and quality of relationship with the hematologist and nurse [19, 55].

Clinical evidence from both China and other countries suggests that, compared with on-demand treatment, even low-dose regimen can significantly reduce bleeding in children with hemophilia, but caused a certain degree of joint damage [26, 60]. With the improvement of economic and medical conditions in the country, a medium or standard dose is suggested, as standard-dose prophylaxis reported better outcomes and prevented progression of joint damage [24, 30]. Similar results were observed with standard dose in China [46]. However, based on availability of dose in China, a low dose of 10 IU/kg of body weight 2 to 3 times a week is still commonly used. An individualized dosing regimen based on age, venous access, hemorrhagic phenotype, PK characteristics, and supply of coagulation factors is the most preferred treatment option if accessible. Since, better health outcomes were reported with less factor consumption in studies using tailored prophylaxis in China and other regions [23, 34, 47]. Overall, even low dose for short-term is recommended in China.

As prophylaxis was initiated only in the last decade in China, tertiary prophylaxis in adult patients with joint damage is commonly observed. Evidences suggest better clinical outcomes in Chinese patients on any type of prophylaxis. In a study by Wu et al., a significant better QoL was observed in prophylaxis treatment group [49]. Leopold II study, compared efficacy of BAY 81–8973 between Chinese and non-Chinese patients, the results showed a comparable ABRs in both groups. Most of the bleeds were mild to moderate and > 90% of the bleeds in Chinese patients required only ≤ 2 infusions which may be due to current clinical practice in China, where low-doses are used for prophylaxis. Patient’s education by HTC lead to increased awareness of prophylaxis and adherence but the access to prophylaxis may be limited in some patients due economic conditions [48]. Nevertheless, there are no clear consensus on adult prophylaxis, but from reports, prophylaxis with any dose for short-term can reduce the number of bleeding episodes and improve the quality of life [12]. HTCCNC in conjugation with WFH monitors hemophilia care in China and encourages the use of standard-dose prophylaxis as a primary treatment option [42, 44].

### Future perspectives

Although the usual treatment recommended for hemophilia A is FVIII replacement therapy, the most challenging complication with the course of treatment is the development of anti-FVIII alloantibodies that affect approximately one-third of patients [64, 65]. Such inhibitors impact patient’s safety and effective treatment, leading to an increased risk of morbidity and mortality, which neutralizes the functional activity of FVIII clotting factor administered with replacement therapy [66]. To overcome these, bypassing agents such as activated prothrombin complex concentrate and activated recombinant FVII are used. In addition, a growing interest in alternative pharmacological therapies that act by enhancing coagulation, such as emicizumab, or inhibiting anticoagulant pathways, such as fitusiran and concizumab, are under clinical investigation [67–69]. WFH guidelines and BSH guidelines recommend Emicizumab prophylaxis [8, 13]. It is not recommended for acute bleeding episodes, for breakthrough bleeds clotting factor concentrates are recommended or bypassing agents in patients with inhibitors [8].

## Conclusion

The results indicate that non-adherence to prophylaxis among young adults transitioning from childhood can lead to high ABRs and joint deterioration. Hence, adherence to prophylaxis is critical among adults. Primary prophylaxis gives better clinical outcomes while secondary and tertiary prophylaxis is better than on-demand treatment. In countries like China, low-dose prophylaxis can help in preventing joint bleeds in the short term compared with on-demand treatment. However, in the long term and with improvement in economic conditions, standard-dose therapy or individualized prophylaxis is suggested as it preserves joint health and improves the quality of life, in turn helping in high adherence to treatment among young adults.

## Supplementary Information

**Additional file 1**. Qualitative assessment of articles included using Newcastle Ottawa scale.

## Data Availability

The data sets generated and/or analyzed during the current study are available from the corresponding author on reasonable request.

## References

[1] Mannucci PM, Tuddenham EG (2001). The hemophilias–from royal genes to gene therapy. N Engl J Med.

[2] Srivastava A, Brewer AK, Mauser-Bunschoten EP (2013). Guidelines for the management of hemophilia. Haemophilia.

[3] Pratap R, Misra M, Morampudi S, Patil A, Reddy J (2019). The existing scenario of haemophilia care in Canada and China—A review. Hematol Trans Cell Therapy.

[4] REPORT ON THE ANNUAL GLOBAL SURVEY 2018. http://www1.wfh.org/publications/files/pdf-1731.pdf

[5] Nordic Hemophilia Guidelines. http://nordhemophilia.org/library/Files/PDF-skjol/NordicGuidelinesCongenitalHaemophilia_2017.pdf

[6] van Dijk K, Fischer K, van der Bom JG, Grobbee DE, van den Berg HM (2005). Variability in clinical phenotype of severe haemophilia: the role of the first joint bleed. Haemophilia.

[7] van Dijk K, Fischer K, van der Bom JG, Scheibel E, Ingerslev J, van den Berg HM (2005). Can long-term prophylaxis for severe haemophilia be stopped in adulthood? Results from Denmark and the Netherlands. Br J Haematol.

[8] Srivastava A, Santagostino E, Dougall A (2020). WFH guidelines for the management of haemophilia, 3rd edition. Haemophilia.

[9] Luck JV, Silva M, Rodriguez-Merchan EC, Ghalambor N, Zahiri CA, Finn RS (2004). Hemophilic arthropathy. J Am Acad Orthop Surg.

[10] Gringeri A, Ewenstein B, Reininger A (2014). The burden of bleeding in haemophilia: is one bleed too many?. Haemophilia.

[11] Ljung R, Fischer K, Carcao M, Santagostino E, Manco-Johnson MJ, Mathew P (2016). Practical considerations in choosing a factor VIII prophylaxis regimen: Role of clinical phenotype and trough levels. Thromb Haemost.

[12] Thrombosis and Hemostasis Group, Chinese Society of Hematology, Chinese Medical Association/Hemophilia Treatment Center Collaborative Network of China. [Chinese guidelines on the treatment of hemophilia (version 2020)]. Zhonghua Xue Ye Xue Za Zhi. 2020;41(4):265–271. 10.3760/cma.j.issn.0253-2727.2020.04.001

[13] Rayment R, Chalmers E, Forsyth K (2020). Guidelines on the use of prophylactic factor replacement for children and adults with Haemophilia A and B. Br J Haematol.

[14] Yang R, Poon M-C, Luke KH (2019). Building a network for hemophilia care in China: 15 years of achievement for the Hemophilia Treatment Center Collaborative Network of China. Blood Adv.

[15] Dargaud Y, Delavenne X, Hart DP, Meunier S, Mismetti P (2018). Individualized PK-based prophylaxis in severe haemophilia. Haemophilia.

[16] Stoffman J, Andersson NG, Branchford B (2019). Common themes and challenges in hemophilia care: a multinational perspective. Hematology.

[17] García-Dasí M, Aznar JA, Jiménez-Yuste V (2015). Adherence to prophylaxis and quality of life in children and adolescents with severe haemophilia A. Haemophilia.

[18] Krishnan S, Vietri J, Furlan R, Duncan N (2015). Adherence to prophylaxis is associated with better outcomes in moderate and severe haemophilia: results of a patient survey. Haemophilia.

[19] De Moerloose P, Urbancik W, Van Den Berg HM, Richards M (2008). A survey of adherence to haemophilia therapy in six European countries: results and recommendations. Haemophilia.

[20] Moher D, Liberati A, Tetzlaff J, Altman DG, PRISMA Group. Preferred reporting items for systematic reviews and meta-analyses: the PRISMA statement. PLoS Med. 2009;6(7):e1000097. 10.1371/journal.pmed.1000097 10.1371/journal.pmed.1000097 PMC2707599 19621072

[21] Khoriaty R, Taher A, Inati A, Lee C (2005). A comparison between prophylaxis and on demand treatment for severe haemophilia. Clin Lab Haematol.

[22] Gringeri A, Lundin B, von Mackensen S, Mantovani L, Mannucci PM, ESPRIT Study Group. A randomized clinical trial of prophylaxis in children with hemophilia A (the ESPRIT Study). J Thromb Haemost. 2011;9(4):700–710. 10.1111/j.1538-7836.2011.04214.x 10.1111/j.1538-7836.2011.04214.x 21255253

[23] Valentino LA, Mamonov V, Hellmann A (2012). A randomized comparison of two prophylaxis regimens and a paired comparison of on-demand and prophylaxis treatments in hemophilia A management. J Thromb Haemost.

[24] Manco-Johnson MJ, Sanders J, Ewing N (2013). Consequences of switching from prophylactic treatment to on-demand treatment in late teens and early adults with severe haemophilia A: the TEEN/TWEN study. Haemophilia.

[25] Oldenburg J, Zimmermann R, Katsarou O (2015). Controlled, cross-sectional MRI evaluation of joint status in severe haemophilia A patients treated with prophylaxis vs. on demand. Haemophilia.

[26] Kavakli K, Yang R, Rusen L (2015). Prophylaxis vs. on-demand treatment with BAY 81-8973, a full-length plasma protein-free recombinant factor VIII product: results from a randomized trial (LEOPOLD II). J Thromb Haemost.

[27] Armstrong EP, Malone DC, Krishnan S, Wessler MJ (2015). Adherence to clotting factors among persons with hemophilia A or B. Hematology.

[28] Tagliaferri A, Feola G, Molinari AC (2015). Benefits of prophylaxis versus on-demand treatment in adolescents and adults with severe haemophilia A: the POTTER study. Thromb Haemost.

[29] Mizrahi T, St-Louis J, Young NL (2016). Adherence to treatment regimen and bleeding rates in a prospective cohort of youth and young adults on low-dose daily prophylaxis for severe hemophilia A. BMC Hematol.

[30] Pérez-Robles T, Romero-Garrido JA, Rodriguez-Merchan EC, Herrero-Ambrosio A (2016). Objective quantification of adherence to prophylaxis in haemophilia patients aged 12 to 25 years and its potential association with bleeding episodes. Thromb Res.

[31] Nijdam A, Foppen W, De Kleijn P (2016). Discontinuing early prophylaxis in severe haemophilia leads to deterioration of joint status despite low bleeding rates. Thromb Haemost.

[32] Schrijvers LH, Beijlevelt-van der Zande M, Peters M, et al. Adherence to prophylaxis and bleeding outcome in haemophilia: a multicentre study. Br J Haematol. 2016;174(3):454–460. 10.1111/bjh.14072 10.1111/bjh.14072 27098446

[33] Manco-Johnson MJ, Lundin B, Funk S (2017). Effect of late prophylaxis in hemophilia on joint status: a randomized trial. J Thromb Haemost.

[34] Feldman BM, Rivard GE, Babyn P (2018). Tailored frequency-escalated primary prophylaxis for severe haemophilia A: results of the 16-year Canadian Hemophilia Prophylaxis Study longitudinal cohort. Lancet Haematol.

[35] Warren BB, Thornhill D, Stein J (2020). Young adult outcomes of childhood prophylaxis for severe hemophilia A: results of the Joint Outcome Continuation Study. Blood Adv.

[36] Miesbach W, Kittler S, Bauhofer A (2020). Long-term analysis of the benefit of prophylaxis for adult patients with severe or moderate haemophilia A. Haemophilia.

[37] Sudevan R, Beenakumari AA, Ganapathy R, Unni M, Vidyadharan G, Sidharthan N (2020). Intermediate Dose Prophylaxis in Adults with Haemophilia: A Clinical Audit from a Resource Limited Setting. Indian J Hematol Blood Transfus.

[38] Zanon E, Tagliaferri A, Pasca S (2020). Physical activity improved by adherence to prophylaxis in an Italian population of children, adolescents and adults with severe haemophilia A: the SHAPE Study. Blood Transfus.

[39] Dover S, Blanchette VS, Wrathall D (2020). Hemophilia prophylaxis adherence and bleeding using a tailored, frequency-escalated approach: The Canadian Hemophilia Primary Prophylaxis Study. Res Pract Thromb Haemost.

[40] Hoefnagels J, Kars M, Fischer K, Schutgens R, Schrijvers L (2020). The Perspectives of Adolescents and Young Adults on Adherence to Prophylaxis in Hemophilia: A Qualitative Study. PPA.

[41] Songnuy R, Seksarn P, Sosothikul D (2020). Prophylactic vs episodic treatment to prevent bleeds and preserve joint function in Thai children with moderate and severe haemophilia A. Haemophilia.

[42] Hua B, Lian X, Li K, Lee A, Poon M-C, Zhao Y (2016). Low-dose tertiary prophylactic therapy reduces total number of bleeds and improves the ability to perform activities of daily living in adults with severe haemophilia A: a single-centre experience from Beijing. Blood Coagul Fibrinolysis.

[43] Wu R, Sun J, Xiao J (2017). A prospective study of health-related quality of life of boys with severe haemophilia A in China: comparing on-demand to prophylaxis treatment. Haemophilia.

[44] Li C, Zhang X, Zhao Y (2017). Long-term efficacy and safety of prophylaxis with recombinant factor VIII in Chinese pediatric patients with hemophilia A: a multi-center, retrospective, non-interventional, phase IV (ReCARE) study. Curr Med Res Opin.

[45] Tang L, Xu W, Li CG (2018). Describing the quality of life of boys with haemophilia in China: Results of a multicentre study using the CHO-KLAT. Haemophilia.

[46] Sun X, Zhuang J, Zhou X, Li H, Liu Z, Sun J (2018). Efficacy of short-term full-dose prophylaxis in adult Chinese patients with severe hemophilia A. Nan Fang Yi Ke Da Xue Xue Bao.

[47] Sun X, Zhuang J, Zhou X, Liu Z, Sun J. Efficacy of Short-term Individualized Prophylaxis Guided by PK and Joint Evaluation in Chinese Adult Patients with Severe Hemophilia A. https://academy.isth.org/isth/2019/melbourne/263882/xueyan.sun.efficacy.of.short-term.individualized.prophylaxis.guided.by.pk.and.html

[48] Yang R, Sun J, Zhao Y (2019). Efficacy and safety of prophylaxis with BAY 81–8973 in Chinese patients with severe haemophilia A enrolled in the LEOPOLD II trial. Haemophilia.

[49] Wu Y, Lu J, Zhou Y (2021). Long-term joint outcomes of regular low-dose prophylaxis in Chinese children with severe haemophilia A. Haemophilia.

[50] Thornburg CD, Duncan NA (2017). Treatment adherence in hemophilia. Patient Prefer Adherence.

[51] Khair K, Lawrence K, Butler R, O’Shea E, Christie BA (2008). Assessment of treatment practice patterns for severe hemophilia A: a global nurse perspective. Acta Haematol.

[52] Wu R, Wu X, Zhang N (2014). Joint disease status of severe and moderate haemophilia patients at the Beijing Children’s Hospital: early onset and rapid increasing severity of arthropathy in 90% of patients by 6 years of age. Haemophilia.

[53] Fischer K (2012). Prophylaxis for adults with haemophilia: one size does not fit all. Blood Transfus.

[54] Duncan N, Shapiro A, Ye X, Epstein J, Luo MP (2012). Treatment patterns, health-related quality of life and adherence to prophylaxis among haemophilia A patients in the United States: treatment patterns, quality of life and adherence. Haemophilia.

[55] Bonanad S, García-Dasí M, Aznar JA (2020). Adherence to prophylaxis in adult patients with severe haemophilia A. Haemophilia.

[56] Fischer K, Van Der Bom JG, Prejs R (2001). Discontinuation of prophylactic therapy in severe haemophilia: incidence and effects on outcome. Haemophilia.

[57] Zhuang J-M, Sun X-Y, Zhou X, Liu Z-Q, Sun J (2018). Prophylactic treatment with low- and intermediate-dose factor VIII in children with severe hemophilia A: comprehensive evaluation of joint outcomes and correlation analysis. Nan Fang Yi Ke Da Xue Xue Bao.

[58] Fischer K, Steen Carlsson K, Petrini P (2013). Intermediate-dose versus high-dose prophylaxis for severe hemophilia: comparing outcome and costs since the 1970s. Blood.

[59] Gamal Andrawes N, Hashem Fayek M, Salah El-Din N, Atef MR (2020). Effect of low-dose factor VIII prophylaxis therapy on bone mineral density and 25(OH) vitamin D level in children with severe haemophilia A. Haemophilia.

[60] Wu Y, Xiao J, Li Z (2019). Study on long-term joint outcome of regular low dose prophylaxis in children with severe hemophilia A with magnetic resonance imaging score. J China Pediatr Blood cancer..

[61] Fischer K, van der Bom JG, Molho P (2002). Prophylactic versus on-demand treatment strategies for severe haemophilia: a comparison of costs and long-term outcome. Haemophilia.

[62] Sahin S, Sadri S, Baslar Z, Ar MC (2019). Osteoporosis in patients with hemophilia: single-center results from a middle-income country. Clin Appl Thromb Hemost.

[63] Hermans C, de Moerloose P, Dolan G (2014). Clinical management of older persons with haemophilia. Crit Rev Oncol/Hematol.

[64] Mannucci PM, Franchini M (2013). Present and future challanges in the treatment of haemophilia: a clinician’s perspective. Blood Transfus.

[65] Franchini M, Mannucci PM (2011). Inhibitors of propagation of coagulation (factors VIII, IX and XI): a review of current therapeutic practice. Br J Clin Pharmacol.

[66] Franchini M, Mannucci PM (2018). Non-factor replacement therapy for haemophilia: a current update. Blood Transfus.

[67] Meeks SL, Batsuli G (2016). Hemophilia and inhibitors: current treatment options and potential new therapeutic approaches. Hematology Am Soc Hematol Educ Program.

[68] Monahan PE (2015). Emerging genetic and pharmacologic therapies for controlling hemostasis: beyond recombinant clotting factors. Hematol Am Soc Hematol Educ Program.

[69] Schmitt C, Adamkewicz JI, Xu J (2021). Pharmacokinetics and pharmacodynamics of emicizumab in persons with hemophilia A with factor VIII inhibitors: HAVEN 1 Study. Thromb Haemost.

