# Supplementary material for: Factor VIII replacement prophylaxis in patients with hemophilia A transitioning to adults: a systematic literature review
Source: Orphanet J Rare Dis. 2021 Jun 26;16:287. doi: 10.1186/s13023-021-01919-w (PMC8236177; doi:10.1186/s13023-021-01919-w)
Supplement: Supplementary file 1 — Additional file 1. Qualitative assessment of articles included using Newcastle Ottawa scale. [file 13023_2021_1919_MOESM1_ESM.docx]

**Supplementary table 1. Qualitative assessment of articles included using Newcastle Ottawa scale**

| **No** | **Author name** | **Year** | **Title** | **Representativeness** | **Selection** | **Ascertainment** | **Demonstration that outcome of interest was not present at start of study** | **Comparability of participants** | **Assessment of outcome with independent blinding** | **Adequacy of follow up** | **Lost to follow up acceptable** | **Total score** |
| --- | --- | --- | --- | --- | --- | --- | --- | --- | --- | --- | --- | --- |
| 1 | Feldman BM | 2018 | Tailored frequency-escalated primary prophylaxis for severe haemophilia A: results of the 16-year Canadian Hemophilia Prophylaxis Study longitudinal cohort | **1** | **1** | **0** | **1** | **0** | **0** | **1** | **1** | **5** |
| 2 | Oldenburg J | 2015 | Controlled, cross-sectional MRI evaluation of joint status in severe haemophilia A patients treated with prophylaxis vs on demand | **1** | **1** | **1** | **1** | **1** | **0** | **0** | **0** | **5** |
| 3 | Khoriaty R | 2005 | A comparison between prophylaxis and on demand treatment for severe haemophilia | **0** | **1** | **1** | **1** | **1** | **0** | **0** | **0** | 4 |
| 4 | Schrijvers LH | 2016 | Adherence to prophylaxis and bleeding outcome in haemophilia: a multicentre study | **1** | **1** | **1** | **1** | **0** | **0** | **0** | **0** | 4 |
| 5 | Mizrahi T | 2016 | Adherence to treatment regimen and bleeding rates in a prospective cohort of youth and young adults on low-dose daily prophylaxis for severe hemophilia A | **1** | **1** | **1** | **1** | **0** | **0** | **1** | **1** | 6 |
| 6 | García-Dasí M | 2015 | Adherence to prophylaxis and quality of life in children and adolescents with severe haemophilia A | **1** | **1** | **1** | 1 | 1 | 0 | 0 | 0 | 5 |
| 7 | Armstrong EP | 2015 | Adherence to clotting factors among persons with hemophilia A or B | 1 | 1 | 1 | 1 | 0 | 0 | 1 | 0 | 5 |
| 8 | Pérez-Robles T | 2016 | Objective quantification of adherence to prophylaxis in haemophilia patients aged 12 to 25years and its potential association with bleeding episodes | 0 | 1 | 1 | 1 | 1 | 0 | 0 | 0 | 4 |
| 9 | Nijdam A | 2016 | Discontinuing early prophylaxis in severe haemophilia leads to deterioration of joint status despite low bleeding rates | 0 | 1 | 1 | 1 | 1 | 0 | 1 | 1 | 6 |
| 10 | Hua B | 2016 | Low-dose tertiary prophylactic therapy reduces total number of bleeds and improves the ability to perform activities of daily living in adults with severe haemophilia A: a single-centre experience from Beijing | 1 | 1 | 1 | 1 | 0 | 0 | 1 | 1 | 6 |
| 11 | Sun J | 2019 | Efficacy of Short- term Individualized Prophylaxis Guided by PK and Joint Evaluation in Chinese Adult Patients with Severe Hemophilia A |  |  |  |  |  |  |  |  |  |
| 12 | Li C | 2017 | Long-term efficacy and safety of prophylaxis with recombinant factor VIII in Chinese pediatric patients with hemophilia A: a multi-center, retrospective, non-interventional, phase IV (ReCARE) study | 1 | 1 | 1 | 1 | 0 | 0 | 0 | 1 | 5 |
| 13 | Tagliaferri A | 2015 | Benefits of prophylaxis versus on-demand treatment in adolescents and adults with severe haemophilia A: The POTTER study | 1 | 1 | 1 | 0 | 1 | 0 | 1 | 1 | 6 |
| 14 | van Dijk K | 2005 | Can long-term prophylaxis for severe haemophilia be stopped in adulthood? Results from Denmark and the Netherlands | 1 | 1 | 1 | 1 | 1 | 0 | 1 | 0 | 6 |
| 15 | Sun J | 2018 | Efficacy of short-term full-dose prophylaxis in adult Chinese patients with severe hemophilia A | 1 | 1 | 1 | 1 | 0 | 0 | 1 | 1 | 6 |
| 16 | Tang L | 2017 | Describing the quality of life of boys with haemophilia in China: Results of a multicentre study using the CHO-KLAT | 1 | 1 | 1 | 1 | 0 | 0 | 0 | 0 | 4 |
| 17 | Manco-Johnson MJ | 2013 | Consequences of switching from prophylactic treatment to on-demand treatment in late teens and early adults with severe haemophilia A: the TEEN/TWEN study | 1 | 1 | 1 | 1 | 0 | 0 | 0 | 1 | 5 |
| 18 | Wu R | 2017 | A prospective study of health-related quality of life of boys with severe haemophilia A in China: comparing on-demand to prophylaxis treatment | 1 | 1 | 1 | 1 | 0 | 0 | 0 | 1 | 5 |
| 19 | Wu y | 2021 | Long-term joint outcomes of regular low-dose prophylaxis in Chinese children with severe haemophilia A | 1 | 1 | 1 | 0 | 1 | 0 | 1 | 1 | 6 |
| 20 | Warren BB | 2020 | Young adult outcomes of childhood prophylaxis for severe hemophilia A: Results of the joint outcome continuation study | 1 | 1 | 1 | 0 | 1 | 0 | 1 | 1 | 6 |
| 21 | Miesbach W | 2020 | Long-term analysis of the benefit of prophylaxis for adult patients with severe or moderate haemophilia A | 1 | 1 | 1 | 0 | 1 | 0 | 1 | 1 | 6 |
| 22 | Sudevan R | 2020 | Intermediate Dose Prophylaxis in Adults with Haemophilia: A Clinical Audit from a Resource Limited Setting | 1 | 1 | 1 | 0 | 1 | 0 | 0 | 1 | 5 |
| 23 | Zanon E | 2020 | Physical activity improved by adherence to prophylaxis in an Italian population of children, adolescents and adults with severe haemophilia A: The SHAPE Study | 1 | 1 | 1 | 0 | 1 | 0 | 1 | 1 | 6 |
| 24 | Dover S | 2020 | Hemophilia prophylaxis adherence and bleeding using a tailored, frequency-escalated approach: The Canadian Hemophilia Primary Prophylaxis Study | 1 | 1 | 1 | 0 | 0 | 0 | 1 | 1 | 5 |
| 25 | Hoefnagels JW | 2020 | The perspectives of adolescents and young adults on adherence to prophylaxis in hemophilia: A qualitative study | 1 | 1 | 1 | 1 | 0 | 0 | 1 | 1 | 6 |
| 26 | Songnuy R | 2020 | Prophylactic vs episodic treatment to prevent bleeds and preserve joint function in Thai children with moderate and severe haemophilia A | 1 | 1 | 1 | 0 | 1 | 0 | 0 | 1 | 5 |
|  |  |  |  |  |  |  |  |  |  |  |  |  |
